# Study on the Mechanical Behavior of Nitrile Rubber Materials Under Thermal-Oil and Thermal–Oxidative Aging in Service Environments

**DOI:** 10.3390/ma19040659

**Published:** 2026-02-09

**Authors:** Jun Wang, Di Chen, Hui Li, Yu Shi, Qiandiao Wei, Bo Cui, Jian Wu

**Affiliations:** 1 Institute for Advanced Materials and Technology, University of Science and Technology Beijing, Beijing 100083, China; wangjun@cei1958.com; 2 State Key Laboratory of Environmental Adaptability for Industrial Products, China National Electric Apparatus Research Institute Co., Ltd., Guangzhou 510663, China; lhui@cei1958.com (H.L.); shiy@cei1958.com (Y.S.); 3 Center for Rubber Composite Materials and Structures, Harbin Institute of Technology, Weihai 264209, China; 25s130173@stu.hit.edu.cn (D.C.); 23b908097@stu.hit.edu.cn (Q.W.); 4 Tibet Guoji Gaoyuan Mechatronics Equipment Scientific Research Co., Ltd., Lhasa 850000, China; 5 Shaanxi HanDe Axle Co., Ltd., Baoji 721000, China; cuibo426@163.com

**Keywords:** nitrile rubber, thermal-oxygen aging, thermal oil aging, mechanical properties

## Abstract

Rubber sealing materials’ aging behavior under challenging circumstances, such as high temperatures, oxygen exposure, or oil immersion, significantly effects how well they seal and how long they last. In order to systematically examine nitrile rubber’s aging behavior and the evolution of mechanical properties under thermo–oxidative and thermo-oil conditions, this study used accelerated high-temperature aging tests. Test results indicate that in a hot-oil environment, the rubber exhibits significant swelling, with mass increasing by up to 9.96%. Hardness undergoes a non-monotonic change, first decreasing and then increasing. In contrast, under thermal-oxidative conditions, hardness increases continuously, exhibiting a marked rise after 7 days of aging at 125 °C. Mechanical property tests revealed a substantial increase in elastic modulus after thermal–oxygen aging. At 125 °C, the modulus rose from an initial 0.4128 MPa to 0.9626 MPa, representing an approximate 133% increase. The compression set reached 83.23% after 7 days of thermal–oxygen aging at 125 °C, compared to 66.89% under thermal-oil conditions. Infrared spectroscopy analysis further indicates enhanced nitro groups and alterations in other functional groups during aging, confirming oxidative chain scission and crosslinking reactions. This study provides quantitative experimental evidence for predicting the service life and optimizing the performance of nitrile rubber under severe environmental conditions.

## 1. Introduction

With the advancement of the rubber industry, rubber materials have been extensively applied across various fields, including aerospace, construction machinery, medical equipment, and automotive electronics [1,2,3,4,5]. During actual service, rubber products undergo mechanical property changes due to environmental influences such as oxygen, humidity, temperature, and light, thereby constraining their service life. Rubber aging refers to the degradation of raw and vulcanized rubber’s composition and structure during storage or use, caused by the combined effects of internal and external factors. This process progressively diminishes the material’s original properties and may even render it unusable [6,7,8,9,10,11,12]. However, under complex service conditions involving high temperatures, high pressures, oxygen, or oil media, rubber materials are prone to irreversible chemical and physical aging [13,14,15,16], leading to diminished mechanical properties, seal failure, and potentially severe safety incidents. Therefore, investigating rubber aging behavior under harsh environments and elucidating its performance evolution patterns holds significant theoretical and engineering importance.

NBR, a polymer formed by emulsion copolymerization of butadiene and acrylonitrile, exhibits not only excellent oil resistance but also wear resistance and aging resistance, making it widely used in various sealing systems [17,18,19]. Currently, research on NBR aging behavior worldwide has evolved from single-factor analyses (e.g., heat, oxygen, moisture, radiation [20,21,22,23,24]) to systematic investigations of multi-field coupling effects. Studies reveal that the aging process of NBR exhibits a strong correlation with the aging environment and involves highly complex, irreversible chemical transformations [25,26,27,28,29,30]. Liu et al. combined macroscopic mechanical behavior changes with microscopic infrared spectroscopy to reveal that when exposed to radiation or high temperature and pressure, the aging mechanism of NBR involves molecular backbone chain scission, oxidation of carbon–carbon double bonds on the backbone, and crosslinking [25,26]. Concurrently, additive molecules within the rubber undergo migration and volatilization [27]. Zhang et al. further employed Fourier Transform Infrared Spectroscopy (FTIR), Scanning Electron Microscopy (SEM), X-ray Photoelectron Spectroscopy (XPS), and Dynamic Mechanical Analysis (DMA) to reveal that thermal oxidation induces dissociation of coordination bonds and increased covalent crosslink density in NBR, leading to rubber embrittlement and deterioration of shape-memory properties [30]. For characterizing and predicting NBR performance during service, combining time–temperature equivalent (TTS) life models with finite element numerical simulations has emerged as an advanced approach for forecasting seal performance degradation and leakage rate evolution [28,31]. Overall, research in this field is advancing toward developing refined, multi-physics coupled predictive models to provide a robust theoretical foundation for seal reliability design, life assessment, and material selection [20,32,33,34,35]. However, to our knowledge, systematic studies on NBR under thermal-oil and thermal–oxygen aging are scarce, leaving a gap in reliable and accurate data support for related research.

This study focuses on nitrile rubber, designing and conducting systematic accelerated aging tests under thermal–oxygen and thermal-oil conditions. The study aims to precisely control aging temperatures (85 °C and 125 °C) and durations (1–7 days) because aircraft fuel systems, hydraulic systems, and environmental control systems in the aviation sector frequently operate at temperatures between 80 °C and 130 °C, with rubber serving as the core sealing material. This study systematically characterizes and comparatively analyzes the evolution patterns of macro-level properties in nitrile rubber under two aging pathways, including the compression set, hardness, uniaxial compression mechanical behavior, and mass change. Concurrently, by integrating surface-morphology observation and infrared-spectroscopy analysis, it investigates the chemical changes in the molecular chain structure during the aging process at the micro-scale, establishing an intrinsic connection between macro- and micro-level properties. This study aims to deepen the understanding of the aging mechanisms of nitrile rubber under different environmental conditions. It provides robust experimental data and theoretical support for the selection optimization, life assessment, and reliability design of rubber sealing materials in engineering practice.

## 2. Materials and Methods

### 2.1. Preparation of NBR Material Test Specimens

The nitrile rubber used in this study was supplied by Xinxiang Haiguang Trading Co., Ltd. (Xinxiang, China) in the form of fully compounded but unvulcanized sheets. Its composition included NBR, zinc oxide, stearic acid, antioxidant D, di-n-butyl sebacate, accelerator NOBS, sulfur, and carbon black as a reinforcing agent. All test specimens were sourced from the same batch to ensure consistency in formulation and filler content throughout the aging process. Prepare test specimens from nitrile sealing rubber according to the national standard GB/T 1683-2018 [36]. Weigh and cut the nitrile compound into small pieces of equal weight, place them into circular cavities of the vulcanization mold, and vulcanize on a plate vulcanizer to obtain rubber specimens. The vulcanization temperature is 150 °C. Once the heating plate reaches the set temperature, place the mold containing the rubber material onto it to commence vulcanization. The vulcanization time is set to 30 min. During the initial vulcanization stage, pressure is applied for 10 s, followed by unloading for 5 s, repeated twice to remove air bubbles within the material and ensure vulcanization quality.

**Figure 1:**
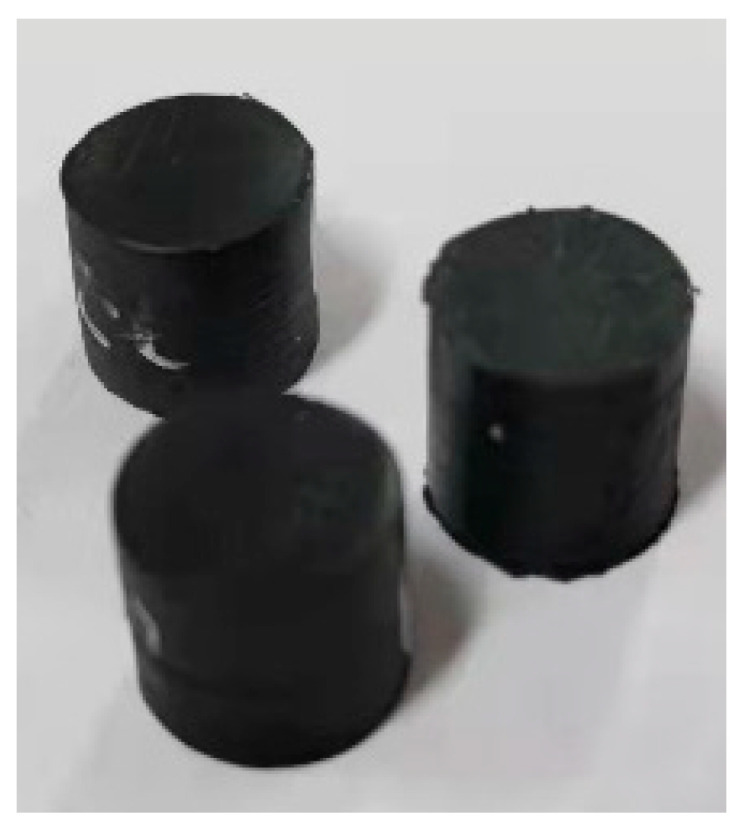
Cylindrical NBR test specimen.

The vulcanized rubber cylindrical specimens were obtained. The specimen diameter is Φ10.0 ± 0.2 mm, height is 10.0 ± 0.2 mm, with a relatively low degree of crosslinking, as shown in Figure 1.

### 2.2. Accelerated Aging

The thermal–oxygen aging test was conducted in a aging constant-temperature chamber (401-A, Yangzhou Daochun Testing Machinery Factory, Yangzhou, China) with a temperature fluctuation of ±1 °C. The selected test temperatures were 85 °C and 125 °C. Hot-oil aging involves immersing test specimens in an oil medium for aging tests under high-temperature conditions. Aviation hydraulic oil No. 15, grade GJB 1177-1991, is used, with a kinematic viscosity of 13.82 mm^2^/s at 50 °C. It is widely employed in aviation hydraulic systems due to its excellent lubricating properties and good compatibility with sealing materials. Aging conditions are detailed in Table 1.

### 2.3. Characteristic Profile

#### 2.3.1. Mechanical Properties Testing

The hardness of rubber materials is typically expressed using Shore hardness, measured with a Shore A hardness tester. Place the specimen on a level test bench, ensuring the hardness tester’s indenter can penetrate the rubber specimen vertically. Press the hardness tester into the rubber specimen’s surface until the presser foot is in full contact with the specimen. Apply a load of 8.05 N and read the value within 1 s. Each specimen is tested five times, and the median value is recorded.

Compressive modulus tests are conducted on rubber sealing materials with varying degrees of aging using uniaxial compression testing. Testing standards comply with ISO 7743:2017 [37]. The testing apparatus used is a tensile/compression testing machine, model UH4204GD, with a range of 0–20 kN, and a maximum tensile (compression) speed of 500 mm/min.

#### 2.3.2. Compression Set

When in use, some rubber items, like seals, frequently experience compression. The product’s quality and performance are directly impacted by its resistance to compression. A rubber’s resistance to compression is typically described using a compression set. When a rubber product is compressed, mechanical stress causes it to alter physically and chemically while in use. Compression set results from the rubber’s inability to completely return to its original state after the compressive force is eliminated. Using the GB/T 1683-2018 [36] test the compression set of sealing rubber at various ages with a recovery time of 30 min.

The compression set rate P is calculated as follows:

(1) $$P=\frac{h_{0}-h_{1}}{h_{0}-h_{2}}\times 100\%$$

where

$h_{0}$—Initial height of the specimen (mm);

$h_{1}$—Height of the specimen after recovery following the test (mm);

$h_{2}$—Height of the limiter (mm).

#### 2.3.3. Surface Morphology Analysis

The surface microstructure of the aged rubber specimens was observed using an optical digital microscope (Model OLYMPUS DSX 510). Prior to observation, the specimen surfaces were cleaned with alcohol to remove grease and contaminants, then air-dried naturally. Observations were then conducted for comparison.

#### 2.3.4. Infrared Spectroscopy

Infrared spectroscopy analysis was performed on specimens before and after aging. The instrumentation was composed of a Nicolet 380 Fourier Transform Infrared Spectrometer from Thermo Electron Corporation (Waltham, MA, USA), operating at a resolution of 4 cm^−1^, with 32 scans, and a wavenumber range of 4000–600 cm^−1^. This FTIR spectrometer performs Fourier transforms on interfered infrared light to generate the material’s infrared spectrum. Analysis was conducted by comparing absorption peaks at various wavenumbers across the spectrum, correlating these with the wavenumber ranges corresponding to specific molecular structures and chemical bonds.

## 3. Results and Discussion

### 3.1. Changes in Mechanical Properties of NBR

#### 3.1.1. Mass Change Patterns

**Figure 2:**
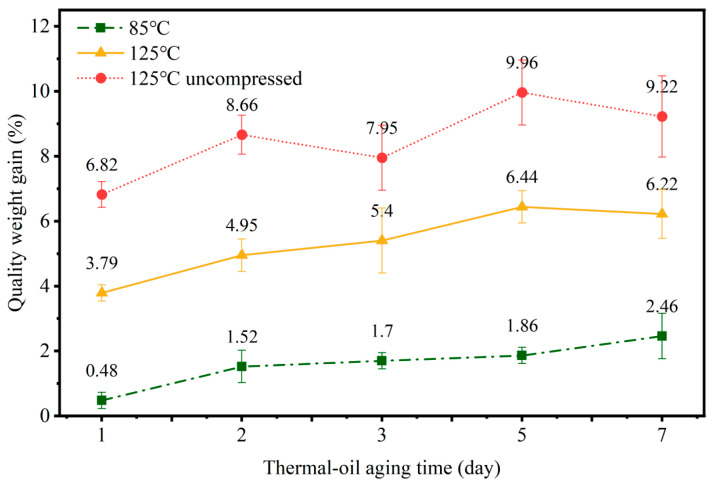
Comparison of NBR mass changes under thermal-oil conditions.

Figure 2 shows the mass change over time for rubber specimens under two thermal-oil aging conditions. At 85 °C, the mass gradually increases with aging days, peaking at 2.46%. At 125 °C, mass initially increases steadily, reaching a maximum of 6.44% after five days of aging before slightly decreasing at seven days. Increased temperature promotes rubber swelling. Additionally, a rubber specimen without compression at 125 °C was introduced as a control. It can be observed that without compressor restriction, the rubber specimen’s mass increased significantly by 6.82% after one day of aging, reaching a maximum increase of 9.96% thereafter without further increase. This indicates that at 125 °C, swelling may reach a certain threshold after five days of aging, preventing further mass increase.

Under thermal–oxygen aging conditions, the mass of the rubber specimens showed minimal change. However, under thermal-oil aging conditions, the mass of the rubber specimens varied with increasing aging time. This is because, under thermal-oil aging conditions, the rubber specimens are fully immersed in the oil medium, which induces several effects on the vulcanized rubber. For example, the rubber absorbs the oil medium; soluble components within the rubber may dissolve out; and chemical reactions may occur between the rubber and the oil medium. Typically, oil absorption by rubber is the most pronounced phenomenon, leading to an increase in rubber volume and mass. This phenomenon is referred to as swelling. After swelling, the physical properties of the rubber, such as hardness and elastic modulus, undergo changes. Furthermore, when submerged in liquid media, some non-mineral additives in rubber materials, such as antioxidants, anti-degradation agents, and strengthening fillers, may dissolve, changing the functionality of rubber sealing materials. Consequently, changes in mass also serve as an evaluation parameter for the performance of rubber sealing specimens.

#### 3.1.2. Patterns of Hardness Change

**Figure 3:**
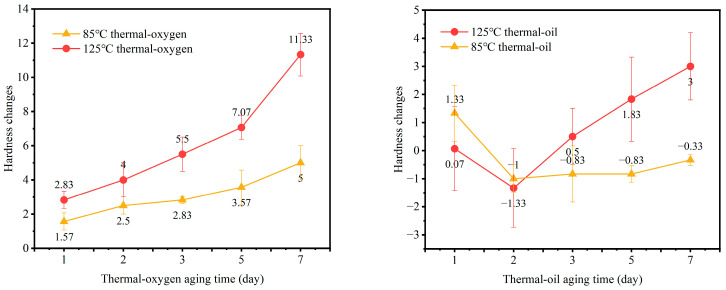
Comparison of hardness changes in NBR under different aging conditions.

Hardness, as a key mechanical parameter for measuring a rubber material’s resistance to external indentation, directly reflects changes in the internal crosslinked network structure and molecular chain mobility. This study systematically examined the temporal evolution of hardness in NBR subjected to thermal–oxygen aging at 85 °C and thermal-oil aging at 125 °C. The specific trends are illustrated in Figure 3. In-depth analysis of these curves reveals the complex physical and chemical transformations occurring within the material under different aging conditions.

Under thermo–oxidative aging, the hardness of rubber specimens exhibited a clear and monotonically increasing trend, closely linked to oxidation-driven chain rearrangement processes. At the relatively mild condition of 85 °C, hardness increased gradually with aging time, reaching a value approximately 5 Shore A units higher than the unaged initial state by the seventh day. This gradual hardening is primarily attributed to sustained thermo–oxidative effects, inducing moderate post-crosslinking reactions. Specifically, new partially rigid carbon–carbon or carbonyl bond crosslinking structures are formed within the existing sulfur-based network, thereby restricting the mobility of molecular segments [38]. However, when the aging temperature is significantly elevated to 125 °C, the hardening process accelerates dramatically, resulting in a hardness increase of approximately 11 Shore A units after seven days. High temperatures substantially enhance oxygen diffusion rates and reactivity within the rubber, causing severe oxidative cleavage of polymer backbones—particularly the unsaturated double bonds in butadiene segments. The resulting free radical fragments rapidly recombine, forming densely packed crosslinked regions. This excessive crosslinking renders the entire rubber network rigid and brittle, manifesting macroscopically as a significant hardness jump.

Compared to the clear trend observed in thermal–oxygen aging, hardness changes under thermal-oil aging conditions exhibit more complex and non-monotonic characteristics. This reflects the dynamic interplay between two competing mechanisms in the oil medium: “swelling” and “aging-induced hardening.” In the thermal-oil environment, specimens initially exhibit a decreasing hardness trend during the early-aging phase. This is due to the swelling effect of the rubber, where oil molecules permeate into the rubber matrix, reducing its hardness. At this stage, the mild crosslinking reactions triggered by heat have only just begun to exert their influence. However, as the aging time increases, the swelling effect of the oil medium on the rubber gradually saturates, while the degree of rubber aging intensifies. The aging–hardening effect then becomes dominant, leading to a gradual increase in rubber hardness. Under the 85 °C hot-oil aging environment, the rubber aging rate is relatively slow. Therefore, during this seven-day experiment, the rubber hardness generally decreased. In the more severe 125 °C hot-oil environment, the competitive process becomes more pronounced and dynamic. During the first two days of aging, the specimens also exhibited a decreasing hardness trend. At this stage, the high temperature significantly accelerates the oil’s penetration and diffusion rate, causing the swelling and softening effect to manifest rapidly. Nevertheless, as aging time extended further, the curve exhibited a clear trend of hardness rebounding from its trough and continuing to increase. The mechanism behind this phenomenon lies in the prolonged high-temperature environment, transforming the oil medium from a simple plasticizer. The oil itself may oxidize and deteriorate, continuously extracting small-molecule additives from the rubber. More critically, the oxidation-driven crosslinking reaction intensifies and accumulates over time. When the “hardening” effect from increased crosslink density due to oxidation begins to outweigh the “softening” effect from oil molecular swelling, the macro-hardness balance shifts toward an increase. At this stage, although swelling persists, its influence is overshadowed by more pronounced chemical aging.

In summary, the evolution of NBR hardness under different aging conditions reflects the dynamic equilibrium of its internal crosslinking network structure under the combined effects of heat, oxygen, oil, and mechanical stress. In a pure thermal–oxygen environment, oxidation-driven chain rearrangement is the sole dominant process, leading to a synchronous, monotonically increasing trend in both hardness and compression set. In a thermal-oil environment, however, hardness changes directly reflect the interplay between oil molecule swelling (tending toward softening) and polymer oxidation crosslinking (tending toward hardening). During the initial aging phase, the swelling effect may dominate. Long-term, especially at elevated temperatures, irreversible chemical aging ultimately dictates the material’s final state, driving it toward hardening. This, coupled with continuously increasing compression set, signals the failure of the material’s sealing function. This principle provides crucial guidance for predicting the service life of rubber seals under complex operating conditions.

#### 3.1.3. Variation Patterns of NBR Elastic Modulus Under Thermo–Oxidative Aging Conditions

**Figure 4:**
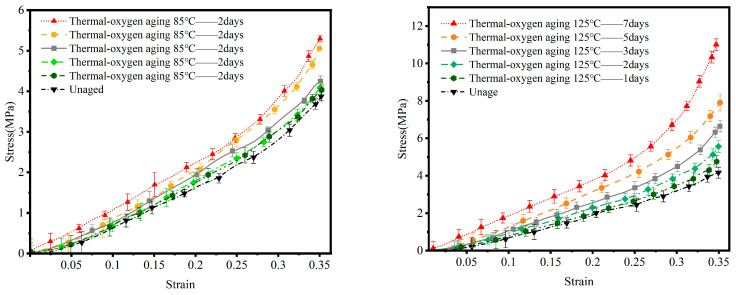
Stress–strain curves of NBR under thermal–oxygen aging at 125 °C and 85 °C.

Figure 4 and Table 2 present the uniaxial compression stress–strain curves and the variation patterns of Young’s modulus for nitrile rubber after thermal–oxygen aging at 85 °C and 125 °C for different durations. Comprehensive analysis of these curves clearly reveals the profound impact of thermal–oxygen aging on the evolution of material stiffness.

Under 85 °C thermal–oxygen aging, material-property changes occur relatively gradually. The stress–strain curves of unaged specimens and those aged for 1 day and 3 days are very similar in the initial stage (strain < 0.25), showing minimal difference in elastic modulus. This indicates that during the early-aging stage or at lower temperatures, the accumulation rate of oxidative crosslinking is slow and has not yet produced a significant global impact on the network structure. However, as aging extended to 5 and 7 days, the curves exhibited a noticeable upward shift. This shift became particularly pronounced during the strain hardening phase beyond 0.25 strain, where the slope increased significantly and the elastic modulus demonstrated a considerable increase.

Under thermal–oxygen aging at 125 °C, the degradation pattern becomes more pronounced and severe. The figure clearly traces a progression: as aging time increases, the stress–strain curves systematically shift upward at nearly equal intervals. Even specimens aged for just 1 day exhibit curves markedly higher than those of unaged specimens. Specimens aged for 7 days exhibited the highest elastic modulus among all samples. Particularly in the high-strain region beyond 0.3 strain, the curve slope steeply increased, indicating the material became abnormally rigid and lost the high elasticity characteristic of rubber. High temperatures significantly accelerate oxygen diffusion rates and the kinetics of oxidation reactions, causing rapid oxidative crosslinking of molecular chains within a short timeframe. This leads to accelerated network structure brittleness, resulting in a sudden increase in macroscopic stiffness over a brief period.

#### 3.1.4. Variation Patterns of NBR Elastic Modulus Under Thermal-Oil Aging Conditions

**Figure 5:**
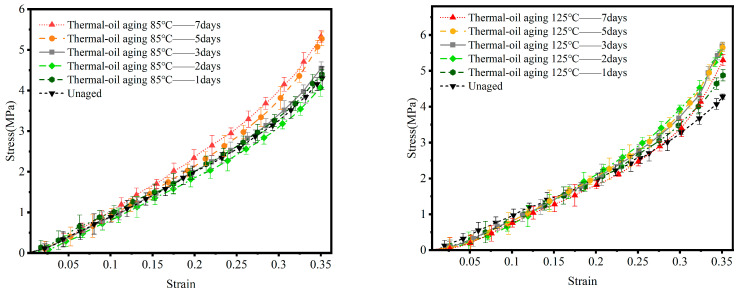
Stress–strain curves of NBR under thermal-oil aging at 125 °C and 85 °C.

Compared to the relatively clear hardening trend observed during thermal–oxygen aging, the uniaxial compressive mechanical behavior of nitrile rubber in a thermal-oil environment exhibits greater complexity and non-monotonicity, as illustrated in Figure 5 and summarized in Table 3. This complexity arises from two competing physicochemical mechanisms present during thermal-oil aging: aging–hardening, caused by oxidative reactions, and swelling–softening, induced by oil molecule permeation.

Under thermal-oil aging conditions at 85 °C, the stress–strain curve exhibits a distinct pattern compared to thermal–oxygen aging. Specimens aged for 1 and 3 days showed no significant upward shift in their curves relative to unaged specimens, and even exhibited a slight downward shift in the initial stage. This indicates that the swelling and softening effect may dominate during the early-aging phase. Oil molecules permeate the rubber network, acting similarly to plasticizers. They increase the intermolecular distance and weaken interchain interactions, thereby softening the material to a certain extent. However, as aging extended to 5 and 7 days, the curves began to shift upward markedly. This reveals that over time, the cumulative “hardening” effect from ongoing oxidative crosslinking reactions began to outweigh the “softening” effect from oil molecule swelling. Ultimately, this manifested as a net increase in stiffness in the macroscopic mechanical properties.

Under thermal-oil aging conditions at 125 °C, this competitive and dynamic equilibrium process exhibits greater subtlety and complexity. Figure 5 reveals a non-monotonic variation pattern: specimens aged for 1 day and 7 days show little difference in Young’s modulus. Specimens aged for 2, 3, and 5 days, however, exhibit markedly higher elastic moduli and stress levels, with their curves positioned at the top. This phenomenon can be interpreted from a kinetic perspective: During the initial aging phase (e.g., days 1–2), elevated temperatures significantly accelerate oil medium permeation, potentially causing the swelling and softening effect to rapidly reach a peak. Concurrently, oxidation-hardening reactions also accelerate synchronously. Within the 2–5 day window, a dynamic equilibrium may be disrupted. For instance, the oil itself may begin oxidizing and deteriorating, reducing its swelling capacity on the rubber, or the rate of oxidative crosslinking may reach an extremely high value. This could temporarily give the hardening effect absolute dominance in the competition, manifesting as the peak modulus value. By the late aging stage (7 days), although oxidative hardening continues, excessive crosslinking may induce microcracks, or swelling and extraction (plasticizer loss) may reach a new steady state, causing the modulus to slightly decline from its peak. This “first increase, then decrease” pattern highlights the complexity of the thermal-oil aging pathway.

### 3.2. Analysis of NBR Aging Properties

#### 3.2.1. Compression Set

**Figure 6:**
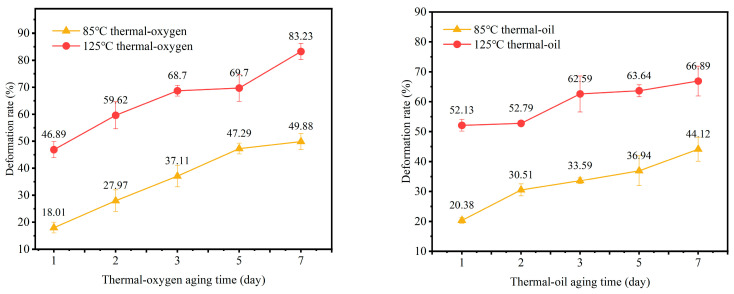
Permanent compression set of NBR under different aging conditions.

As shown in Figure 6, the permanent compression deformation rate of NBR varies under two distinct aging conditions. Comparing the two graphs reveals that both temperature and aging medium jointly govern the evolution of NBR’s compression set. Under thermal–oxygen aging, the aging behavior exhibits typical brittle degradation characteristics: the deformation rate surges dramatically at 125 °C, reaching approximately 83.23% in just 7 days—significantly higher than the 49.88% observed at 85 °C. This indicates that high temperatures greatly accelerate the oxidative breakage of rubber molecular chains, leading to a rapid loss of elasticity and swift failure of sealing functionality. In contrast, under thermal-oil aging conditions, the increase in the deformation rate is relatively moderate. At 125 °C, the deformation rate after 7 days is approximately 66.89%, which is higher than the 44.12% observed at 85 °C. However, both the absolute value and the rate of increase are significantly lower than those under thermal–oxygen aging at the same temperature. This is primarily due to a competitive mechanism formed in the thermal-oil environment: on one hand, oil temperature promotes material aging and hardening, similar to thermal–oxygen aging; while oil molecules infiltrating the rubber network may induce swelling, partially offsetting permanent deformation caused by compression. Notably, the sustained increase in compression set under thermal–oxygen aging exhibits a strong intrinsic correlation with the previously observed sharp rise in hardness. Both phenomena point to the same underlying cause: the core of rubber elasticity—the flexibility and recovery capacity of molecular chains—is severely compromised by oxidative embrittlement of the network structure. The material’s difficulty in rebounding after compression (high deformation rate) and its enhanced resistance to indentation (high hardness) both reflect the same aging mechanism manifested in different mechanical tests.

#### 3.2.2. Surface Morphology Evolution Analysis

Macroscopic property changes in materials often originate from alterations in their microstructure. Table 4 presents a comparison of surface microtopographies for unaged specimens, specimens aged for 7 days at 125 °C under thermal–oxygen conditions, and specimens aged for 7 days at 125 °C under thermal-oil conditions. It should be noted that the visible parallel striations on the specimen surfaces are mechanical marks imprinted by the texture of the tester surface during the compression set test. These are fixed features introduced during the experimental process and are not caused by material aging. They can be disregarded in the analysis.

Comparing the three images above, it is evident that the surface morphology of rubber subjected to thermal–oxygen aging exhibits minimal changes, whereas rubber treated with thermal-oil aging shows severe damage. This occurs because during thermal–oxygen aging, rubber primarily undergoes the combined effects of heat and oxygen, triggering oxidative degradation and crosslinking reactions within the molecular chains. This typically results in the formation of a hardened, brittle oxidation layer on the surface, which gradually develops fine, dense cracks [39]. However, since degradation primarily occurs internally and lacks a mechanism to remove degradation products, the pits formed on the surface are relatively fewer and shallower. Additionally, due to the relatively short aging time in this test, cracks had not yet fully formed and are therefore not visible in the images.

In thermal-oil aging, beyond the effects of heat and oxygen, the oil medium plays a pivotal role. Oil not only permeates and swells rubber, accelerating oxidation, but also continuously dissolves and extracts small-molecule components like plasticizers and accelerators, along with degradation products, from within the rubber. This physical “hollowing-out” effect directly creates numerous microscopic voids. As these voids coalesce and expand, they form dense pitting on the surface. Consequently, thermal-oil aging represents a synergistic amplification of physical erosion and chemical degradation, resulting in far more severe and coarse surface morphology damage than simple thermal–oxygen aging.

#### 3.2.3. Infrared Spectroscopy Analysis of Molecular Structural Changes

**Figure 7:**
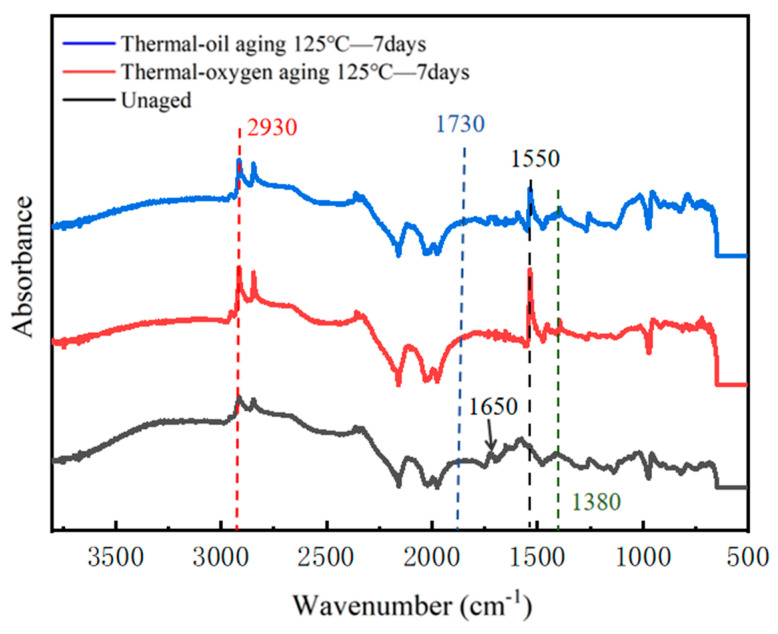
Infrared spectra of NBR specimens before and after aging.

While surface morphology observation reveals the macroscopic consequences of aging, tracing its fundamental causes requires delving into the molecular level. To this end, Fourier Transform Infrared Spectroscopy (FTIR) analysis was conducted on unaged specimens, specimens aged for 7 days under thermal–oxygen conditions, and specimens aged for 7 days under thermal-oil conditions. The results clearly revealed the evolution of chemical bonds and functional groups during the aging process, as shown in Figure 7. Comparing the spectra of the three groups of specimens reveals significant changes in the intensity, shape, and even presence of absorption peaks at multiple characteristic wavenumbers. This directly confirms that the aging process induces chemical transformations in the rubber molecular chains.

The most prominent change occurs near the wavenumber 1550 cm^−1^. The absorption peak at this location corresponds to the stretching vibration of the nitro (-NO_2_) group. In the spectrum of the unaged sample, this peak is extremely weak or nearly indistinguishable. However, after 7 days of aging under either thermo–oxygen or thermo-oil conditions, the characteristic absorption peak at this position significantly intensified. Since the nitro group is not a primary component of the original molecular structure of nitrile rubber, its appearance and enhancement provide direct evidence of nitrogen-related oxidation reactions occurring in the rubber molecular chains under aging conditions. The changes observed under thermal–oxygen aging are particularly pronounced. This may stem from complex oxidation and nitration reactions occurring in the rubber molecular chains (especially in segments containing acrylonitrile) under the combined attack of high temperature and oxygen, leading to the formation of nitro-containing oxidation products [28]. The marked intensification of this peak indicates that oxidative degradation reactions have profoundly altered the fundamental chemical structure of the rubber.

Additionally, the peak at 1650 cm^−1^ has disappeared. This corresponds to the stretching vibration peak of the -C=C- group. Peak shifts also occur at 1730 cm^−1^, 1380 cm^−1^, and 2930 cm^−1^. These correspond to the characteristic C=O peak, the -CH_3_ methyl stretching vibration peak, and the -CH_2_- stretching vibration peak, respectively. Upon aging, the characteristic peaks of -CH_3_ and -NO_2_ exhibit increased intensity. In contrast, the peaks of -C=C- and C=O double bonds weaken. The initial radicals generated under thermal action combine with oxygen. They attack the most reactive carbon–carbon and carbon–oxygen double bonds in the polymer chain. These bonds correspond to the weakened peaks at 1650 cm^−1^ and 1730 cm^−1^. This reaction leads to substantial depletion through addition or cleavage reactions. Simultaneously, decomposition of the generated peroxides triggers double bond cleavage. This process produces abundant methyl end groups. The newly formed methyl end groups, corresponding to the enhanced -CH_3_ peak at 1380 cm^−1^, directly demonstrate the degradation of molecular chains and the reduction in molecular weight [40].

These chemical changes collectively indicate the oxidative cleavage and re-crosslinking of molecular chains, resulting in an increased crosslink network density and reduced segmental mobility. This manifests macroscopically as a persistent increase in hardness, a significantly elevated elastic modulus, and a markedly heightened compression set—characteristics of brittle aging that become particularly pronounced under thermal-oxidative aging conditions.

## 4. Conclusions

Through systematic experiments, this study reveals the aging mechanisms and performance evolution patterns of NBR under thermo–oxygen and thermo-oil environments. The results indicate distinctly different aging pathways in each environment: under thermal–oxygen conditions, aging is primarily driven by oxidative crosslinking reactions, resulting in significant increases in material hardness, elastic modulus, and compression set, exhibiting rapid embrittlement characteristics. In contrast, under thermal-oil conditions, a competitive mechanism exists between oil-induced swelling/softening and oxidative hardening, resulting in complex, non-monotonic changes in macroscopic mechanical properties. Microstructural analysis further confirms that the depletion of carbon–carbon double bonds in molecular chains and the generation of oxygen-containing functional groups are the fundamental causes of material degradation. Additionally, this study elucidates the complete chain from molecular structural changes to macro-performance decline, clarifying that high-temperature thermal oxidation represents the most severe service condition for NBR. This provides a solid theoretical foundation and experimental data for predicting the service life and optimizing the selection of related sealing materials.

## Figures and Tables

**Table 1:** Aging test conditions.

Test Condition	Medium Environment	Temperature (°C)	Aging Duration (Days)
Parameters	Oxygen/Hydraulic Oil	85, 125	1, 2, 3, 5, 7

**Table 2:** Changes in Young’s modulus of nitrile rubber under thermal–oxidative aging conditions.

85 °C Aging Days	Young’s Modulus (MPa)	125 °C Aging Days	Young’s Modulus (MPa)
unaged	0.4128 ± 0.021	unaged	0.4128 ± 0.021
1	0.4219 ± 0.023	1	0.4991 ± 0.028
2	0.4573 ± 0.025	2	0.4991 ± 0.028
3	0.5140 ± 0.029	3	0.6424 ± 0.036
5	0.5143 ± 0.028	5	0.8016 ± 0.042
7	0.5337 ± 0.031	7	0.9626 ± 0.050

**Table 3:** Changes in Young’s modulus of nitrile rubber under hot-oil aging conditions.

85 °C Aging Days	Young’s Modulus (MPa)	125 °C Aging Days	Young’s Modulus (MPa)
unaged	0.4128 ± 0.021	unaged	0.4128 ± 0.021
1	0.4399 ± 0.024	1	0.4316 ± 0.023
2	0.4027 ± 0.022	2	0.4645 ± 0.026
3	0.4422 ± 0.024	3	0.4489 ± 0.025
5	0.4769 ± 0.026	5	0.4562 ± 0.026
7	0.4959 ± 0.027	7	0.4067 ± 0.023

**Table 4:** Rubber surface morphology under different aging conditions.

Unaged	7 Days at 125 °C Thermal–Oxygen Aging	7 Days of 125 °C Thermal-Oil Aging
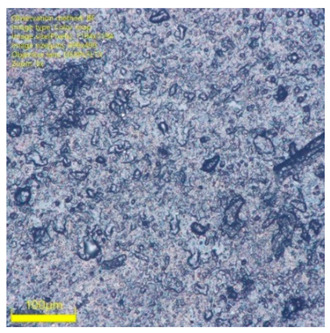	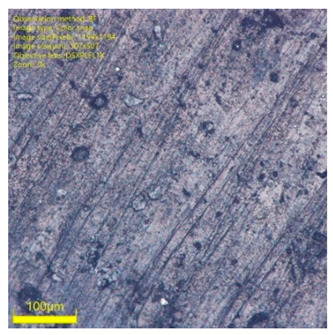	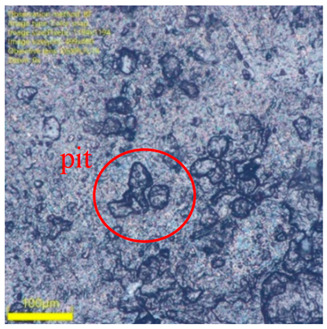

## Data Availability

The original contributions presented in this study are included in the article. Further inquiries can be directed to the corresponding author.
